# A chain mediation model reveals the association between mindfulness and depression of college students

**DOI:** 10.1038/s41598-023-43984-0

**Published:** 2023-10-06

**Authors:** Junliang Zhang, Zhongzheng Hu, Shuang Zheng, Yingyu Liu

**Affiliations:** Science and Technology College, Nanchang Hangkong University, Jiujiang, 332020 China

**Keywords:** Psychology, Health care

## Abstract

The study aims to explore the relationship among mindfulness, depression, meaning in life (MIL) and psychological resilience (PR), and verify whether MIL and PR have a chain mediating effect between mindfulness and depression. 1034 college students in a province were investigated by using Adolescent Mindfulness Scale, Depression Scale, Meaning in Life Scale and Psychological Resilience Scale. Hierarchical multiple regression and bootstrap was used to analyze the data and test whether MIL and PR play a chain mediating role between mindfulness and depression among university students. In the present research, the direct effect value of mindfulness on college students’ depression was 0.14, and the effect amount was 61%. The effect values of MIL and PR between mindfulness and college students’ depression were 0.03 and 0.05, and the effect amount is 13% and 22%. In the meantime, the chain mediating effect of MIL and PR was significant, the effect value is 0.01 and the effect amount is 4%. The effects of mindfulness on depression of university students were mediated by MIL, PR and the chain of MIL and PR.

## Introduction

According to the WHO, as of January 2020, depression has become the second most common disease in the world, affecting 260 million people worldwide. The amount of college students in China as the world’s largest country suffering from depression is increasing on a yearly basis. As of now (May 2023), a research study in China found that the rate of depression detection among Chinese college students was 31%, with a rate of major depression of 8%^1^. Depression has been a common problem among adolescents, which has a range of symptoms that affect physical, cognitive, emotional and social processes^2^. Depression not only has an impact on one’s own physical health, but also adversely affects college studies and social functions, and even causes suicidal ideation and behavior. College students are a high prevalence of depression and its harmfulness, which has drawn the high attention of the Chinese Health Care Commission. Therefore, to explore the influencing factors and working mechanism of college students’ depression is of certain practical significance for reducing and preventing the occurrence of depression and promoting their mental health improvement.

### From mindfulness to depression

Mindfulness is a state of consciousness about one’s heart and the focus of one’s attention at the moment^3^, that is, attending to the present time and being aware of one’s thoughts and sentiments without interference or judgment. Mindfulness is a fundamental human ability that allows us to be fully aware of where we are and what we are doing, rather than overreacting or being overwhelmed by what is happening around us. Mindfulness can support, comfort and enrich our lives and enhance motivation for individual development^4^. Originating from traditional Eastern Buddhism and other meditations, mindfulness has long been regarded as a characteristic of consciousness that is conducive to individual health^5^. Despite its ancient roots, mindfulness is now being used daily in many industries, including business, education and healthcare. Over the past three decades, mindfulness has become one of the most widely used health promotion applications in the West, so much so that words like yoga and meditation are now standard household language^6^. Many studies have shown that mindfulness as a way to help people manage stress is becoming more and more popular. For example, studies have indicated students with higher level of mindfulness perform better in cognitive flexibility and concentration^7^ and are able to possess higher sense of contentment in interpersonal relationship^8^ and lower sense of perceived pressure^9^. The improvement of mindfulness meditation or mindfulness training techniques can reduce the occurrence of depression, or reduce the non-rational factors such as rumination and negative cognition that are prone to depression^10^. Of course, it has also been suggested that the magnitude of trait mindfulness’s effect on depression may in many cases depend on the sample and how it is measured^11^. By using the method of meta-analysis, study found that mindfulness-based therapy (MBT) improved the level of mindfulness, reduced the level of psychological stress and depression^12^. It effectively relieve anger, impulsivity and negative emotions, and increases self-esteem as well. It can be said that mindfulness has been applied to different populations and has had positive effects on reducing his or her physical and mental health^13^. Thus, we propose this hypothesis:

#### H1

Mindfulness has a significant predictive effect on depression in college students.

### Meaning in life as a mediator

The MIL implies the extent to which people understand, give or view the MIL and have goals, missions or primary purposes in life^14^. Sense of MIL has an significant influence on individual physical and mental health. People with low sense of MIL are prone to anxiety, depression and other negative emotions, and even suicidal tendencies, drug abuse and other forms of painful behaviors^15^. People with a high MIL have high positive psychology of being willing to work, life satisfaction and happiness index^16^. Ariely et al.^17^ pointed out that motivation is the most important and common way to obtain a MIL, and individuals without a MIL are characterized by boredom, despair and depression. The model of self-determination theory believes that mindfulness is positively correlated with MIL, and authentic self-awareness components play a moderating role between mindfulness and MIL^18^. Researchers have proved that mindfulness was positively correlated with MIL, and that mindfulness alleviated the frustration of marginalized people to a certain extent and improved MIL through mixed method^19^ Yang et al.^20^ took 764 students as samples and adopted self-report method, and found that studying mindfulness can effectively alleviate depression, and found that MIL and boredom tendency play a moderating and mediating role respectively. Chu et al.^21^ carried out a randomized controlled trial analysis of 7895 subjects, and found that the randomized control group with basic mindfulness intervention could enrich the MIL, and had a reasonable impact on the MIL. Hence, the following hypothesis is established:

#### H2

MIL plays a mediating role in the relationship between mindfulness and depression in college students.

### Psychological resilience as a mediator

PR could be defined as the capability to adjust or change effectively when confronting adversity, which is a pressure protection mechanism^22,23^. Due to individual differences or different reaction strategies in the face of pressure or negative events, people with high PR can easily extricate themselves from stressful events or emotions, while people with low PR need a long time to extricate themselves, even lifelong^24^. As an important variable affecting mental health, PR is also very important to reduce or prevent negative psychology caused by COVID-19^25^. Compared with people with mood disorders, people in good health are more PR, which reduces not only the risk of depression, but also the risk of other mental disorders^26^. Other researches have revealed that mindfulness and PR are negatively correlated with both anxiety and depression, and that mindfulness alleviates the negative impact of learning stress and depression^27^. More and more researches concentrate on the relationship between PR, mindfulness and mental health, indicating that PR and mindfulness are important variables to predict mental health. PR and mindfulness can not only maintain an individual’s physical and mental health, but also enable individuals to recover from negative emotions and stressful events^28,29^. Mindfulness can improve mental state and PR, and research has shown a positive correlation between the two^30^. Therefore, the following hypothesis was developed:

#### H3

H3: PR is a mediator between mindfulness and depression among university students.

### The role of MIL and PR

Through the above literature analysis, it can be seen that MIL and PR play an important role. But how does MIL affect the depression of college students through resilience? Empirical studies show that those with a high MIL have strong PR and are more able to navigate in the face of difficulties. At the same time, some studies have shown that MIL can indeed improve the PR of college students, and it can also play a protective role in adversity^31^. Tay and Lim^32^ found that MIL can reduce or mitigate depression in college students through its positive effect on PR. Similarly, some studies have found that MIL, as an important predictor of PR, can indeed play a positive predictive role^33^. When individuals suffer from anxiety and depression, resilience acts as a protective factor to enhance and resist mental illness.

Studies have shown that people with strong PR usually use effective method to deal with problems. They normally have a clear grasp of reality, a well-established belief system, the ability to adjust oneself quickly to changing conditions, and a strong confidence in the future^34^. The MIL helps individuals with strong PR to more actively cope with and adapt to external difficulties and effectively balance negative and positive emotions Folkman^35^. Recent research results show that people with strong PR tends to discover the MIL in difficult times. More interestingly, PR and MIL have been considered as an important predictors of post-traumatic growth, especially in coping with anxiety, depression and stress^36,37^. There are also studies that suggest PR is an important positive resource that can help individuals find deeper MIL^38^. Hence,the following hypothesis is formulated:

#### H4

H4: MIL and PR play a chain mediating effect between mindfulness and depression in college students.

## Research methods

### Sample

Several universities in Jiangxi, China, 1100 participants were selected for group administration of the test in a class setting by cluster random sample method. First, the guideline of the questionnaire, the purpose of the study, the test format and anonymity were read to the subjects; second, all subjects were asked again if they heard the answer requirements clearly and then completed all questions within the time specified online. All of the participants signed an informed consent form before the test, with mean age of 20.53 years and SD of 0.84. They would be paid after the test. 1034 valid questionnaires were recollected, with an effective questionnaire rate of 94%. This study was approved by the Academic Committee of Science and Technology College of Nanchang Hangkong University. All methods were performed in accordance with relevant guidelines and regulations. Socio-demographics of the total sample is displayed in Table 1.

**Table 1:** Socio-demographics of the total sample.

		N	%
Gender	Male	606	58.6
	Female	428	41.4
Location	Rural area	663	64.1
	Urban area	371	35.9
GRADE	Freshman	402	38.9
	Sophomore	221	21.4
	Junior	210	20.3
	Senior	201	19.4

### Measurements

#### Adolescent mindfulness level scale

This study adpoted Adolescent Mindfulness Level Scale compiled by Greco et al.^39^. The Likert scale has 10 items with a 5-point range from “never” to “constantly,” (for example, “I feel annoyed by some of my ideas.”) All choices are also counted backward. As a result, it was revealed that students’ levels of mindfulness increased in direct proportion to their scores. The Cronbach’s alpha reliability score was 0.84.

#### Meaning in life scale

Wang Xinqiang^40^ compiled the Meaning in Life Scale ,which has 10 items (e.g. “I always try to find the purpose of my life”),using 7-point Likert ranging from “Completely disaccord” to “very accord”, including two dimensions of meaning possession and meaning seeking. Each dimension includes 5 questions respectively, among which the second is “reverse score”. The higher the score is, the higher of MIL is. In actual measurement, the final Cronbach’s alpha was 0.87.

#### Psychological resilience scale

The Psychological Resilience Scale was developed by by Block and Kreman^41^. The scale has 14 items (e.g., “I am considered to be a very energetic person”). Respondents indicate their agreement on a 7-point Likert scale from “not at all” to “completely”),with higher scores indicating better levels of the PR. In the current sample, Cronbach’s alpha indicated satisfying internal consistency 0.90.

#### Self-rating depression scale

Participants’ depression was scored by the Self-rating Depression Scale 42, including a 20-item questions (e.g.,“I don’t sleep well at night “). These items using a four-point Likert scale are scored on a range from “no or very time” to “always”, with higher scores indicating higher depression. In actual measurement, Cronbach’s alpha indicated internal consistency (0.83).

### Statistical analyses

First,descriptive analysis and correlation analysis of each variable were conducted by using the SPSS24.0. The second step is to use the SPSS macro PROCESS Model 6 with 5000 resamples to build the model. In this model, mindfulness was used as an independent variable, depression as a dependent variable, and PR and MIL as mediating variables. Finally, deviation correction Bootstrap method (random sampling 10,000 times) was applied to the Conditional indirect effects of active MIL on PR.

## Result

### Common method bias test

In view of the fact that the data of all subjects in the current study were collected using self-rating scales, there may be common method bias43, so Harman single factor method was used to test them. After the main analysis, 8 characteristic factors were found to be 1. The results showed that the first common factor accounted for 23.47% of the variation, and the critical criterion was less than 40%, indicating that common method bias was not found in this study.

### Bivariate correlations of the key variables

After controlling grade and gender variables, the results of partial correlation analysis indicated that mindfulness was positively correlated with both MIL (*γ *= 0.09, *P* < 0.01) and PR (*γ *= 0.22, < *P* 0.001), and significantly negatively correlated with depression *β* (*γ *= − 0.36, *P* < 0.001); Likewise, MIL was positively correlated with PR (*γ *= *0.58*, *P* < 0.001). In addition, depression of college students was negatively and strongly correlated with both MIL (*γ* = − 0.46, *P* < 0.001) and PR (*γ* = − 0.46, *P* < 0.001). The average value, standard deviation and Pearson correlation coefficient of the main variables in the current study are illustrated in Table 2.

**Table 2:** Correlation analysis results of each variable (N = 1034).

	1	2	3	4	M	SD
1. MQ	1				36.71	7.46
2. MIL	0.09**	1			46.74	10.59
3. PR	0.22***	0.58***	1		38.80	8.17
4. SDS	− 0.36***	− 0.46***	− 0.46***	1	40.91	8.45

*MQ* Mindfulness, *MIL* Meaning in life, *PR* Psychological resilience, *SDS* Depression.

*p < 0.05, **p < 0.01, ***p < 0.001.

### Mediation analyses

**Figure 1:**
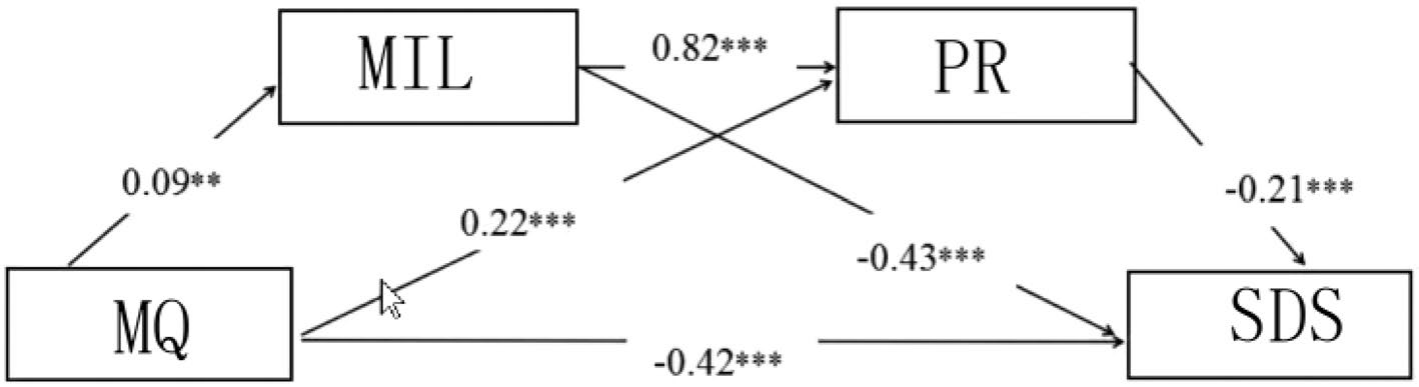
The chain mediation model of college students’ meaning in life and psychological resilience.

In our analysis, Stepwise regression equations was used to test the feasibility of each hypothesis, so the findings were as follows:Mindfulness significantly negatively predicted depression in college students (= *β* − 0.42, *P* < 0.001), hypothesis test H1 was true, and mindfulness significantly positively predicted MIL and PR (*β* = *0.09*, *P* < 0.01;*β* = 0.22, *P* < 0.001); MIL significantly positively predicted PR (*β* = *0.82*, *P* < 0.001), negatively predicted depression (*β* = − *0.43*, *P* < 0.001), PR significantly negatively predicted depression (*β* = − 0.21, *P* < 0.001). The path coefficients in the variables are significantly established, and the hypothesis model is preliminary established (to see Fig. 1).

Furthermore,on the basis of the structural equation, the SPSS macro PROCESS Model 6 with 5000 resamples was further used to exam the chain mediation effect of MIL and PR. The indirect effect of mindfulness on depression through MIL was significant (*β* = − 0.03, 95% CI − 0.08, − 0.002). The mediation effect (MQ → MIL → SDS) accounted for 13% of the total effect. At the same time, PR mediated the relationship between mindfulness and depression (*β *= − 0.05, 95% CI − 0.73, − 0.25). The mediation effect (MQ → PR → SDS) accounted for 22% of the total effect. Lastly , the indirect effect of mindfulness on depression through MIL and PR was also found(*β* = − 0.01, 95% CI − 0.03, − 0.0006). The mediation effect (MQ → MIL → PR → SDS) take up 4% of this total effect. Therefore, the chain mediating effect of MIL and PR was further confirmed. The mediating effect values of each variable are shown in Table 3.

**Table 3:** Bootstrap mediating effects of meaning in life and psychological resilience.

Model pathways	Effect	Boot LLCI	Boot ULCI	Effect of the amount
MQ → SDS	− 0.14	− 0.32	− 0.02	
MQ → MIL → SDS	− 0.03	− 0.078	− 0.002	0.13
MQ → PR → SDS	− 0.05	− 0.73	− 0.25	0.22
MQ → MIL → PR → SDS	− 0.01	− 0.0324	− 0.0006	0.04
Total effect	− 0.09	− 0.16	− 0.04	

## Discussion

To investigating the internal mechanism and individual differences of the effects of mindfulness on depression among university students, our study constructed a chain mediation structure to analyze this chain-mediated effects of MIL and PR on the relationship between mindfulness and depression among college students. This study not only helps to reveal the influence of mindfulness on depression and its internal mechanism, but also provides empirical evidence and suggestions for preventing depression and maintaining mental health of college students.

With the change of living environment, after entering university, in addition to the challenge of life adaptation, college students face the test the pressure of study, which is very easy to produce anxiety, depression and other negative emotions. Depression has become a public health problem of mental health on college campuses, with serious cases of academic failure, dropout and even suicide^44^. This study shows that mindfulness negatively predicts depression of college students. Accordingly, we should make much account of the training of college students’ mindfulness training skills and improve their awareness and level of mindfulness training. The objective of our study is to construct a new frame of reference for the prevention of depression among college students by introducing the variables of meaning in life and PR to establish a chain mediation model.

In this paper, we found that MIL and PR played a chain mediating role in the relationship between mindfulness and depression in college students. The mediating effect value accounts for 39% of the total effect. That is, on the one hand, mindfulness can directly affect the depression level of college students; on the other hand, it can indirectly affect the depression mood of college students through the sense of MIL and PR. First of all, the mediating effect of PR is the largest in the relationship between mindfulness and depression in university students, which indicates that mindfulness alleviates depression mainly by improving the level of mental resilience, which also confirms hypothesis 3 of this study. According to the theory of expansion and construction of positive emotions, negative emotions focus people’s attention on supporting specific action tendencies (such as attack and escape), while positive emotions may inhibit self-awakening caused by negative emotions and expand people’s attention and cognition and behavior ability^45^. Multiple studies have proved that PR is characterized by positive emotions and can help individuals recover from stressful and depressed situations^46,47^.

Secondly, the mediating effect of MIL on the relationship between mindfulness and depression in college students was 0.09, indicating that the hypothesis H2 of this study is valid, and further confirming that sense of life has a good effect on alleviating or inhibiting depression of college students. Frank pointed out that the sense of MIL is the most common and important motivation for human beings. Without it, individuals will become bored, disappointed, depressed and even lose the meaning of existence^48^. Of course, some studies suggest that a person without a sense of MIL is unlikely to be satisfied with life and may even suffer from severe depression^49^. In conclusion, seeking a sense of = MIL can buffer the relationship between depression and anxiety, while playing a vital role in maintaining both the physical and psychological aspects^50^.

Thirdly, this study found that the chain mediation of mindfulness indirectly affecting college students’ depression through MIL and PR was established, and the hypothesis H4 was established. Although the mediating amount is 0.03, which takes up a relatively low percentage of the total effect, it does not mean that the chain effect is meaningless. These results indicate that mindfulness can influence depression in college students through MIL and PR. In other words, the improvement of mindfulness can increase young students’ perception of the MIL, enhance their sense of PR, and thus alleviate or reduce their depression.

Through the mediating test, our study found that there was indeed a chain mediating effect between MIL and PR. Mindfulness not only has a direct impact on depression, but also indirectly has a positive impact on depression and improves positive emotions through MIL and PR. University students are encouraged to train mindfulness skills in their daily lives, establish good MIL values and improve their PR to resist pressure. This study constructs a new theoretical model with college students as the research object, and further deepens the mechanism of mindfulness on depression, which provides guidance and suggestions for the intervention of college students’ depression.

The study has some limitations = , which might to be improved in the future. Firstly, these subjects of the study are all from universities in a certain province, so the generalization of the research conclusion needs further testing and verification. Secondly, in the study we used the cross-sectional study, which could not discuss the long-term effects between variables, and longitudinal studies were considered in the future. Finally, the data are collected from subjective reports of the subjects, and there will be certain errors, so we can consider obtaining data from other aspects.

## Conclusion

The results support our hypothesis that high and low levels of mindfulness do contribute to depression in young students. The higher the individual’s sense of mindfulness, the stronger their ability to resist depression. At the same time, we introduce the sense of MIL and PR as intermediate variables in the structural model to test whether the two variables have a chain mediating effect between mindfulness and depression variables. In fact, this study also proves this hypothesis.

## Data Availability

We agreed to share the data from this study and request it from the corresponding author if necessary.
